# Single-stranded circular DNA theranostics

**DOI:** 10.7150/thno.66466

**Published:** 2022-01-01

**Authors:** Tingting Shen, Yu Zhang, Lei Mei, Xiao-Bing Zhang, Guizhi Zhu

**Affiliations:** 1 Molecular Sciences and Biomedicine Laboratory, State Key Laboratory for Chemo/Biosensing and Chemometrics, College of Chemistry and Chemical Engineering and College of Biology, Collaborative Innovation Center for Molecular Engineering and Theranostics, Hunan University, Changsha 410082, China.; 2 Department of Pharmaceutics and Center for Pharmaceutical Engineering and Sciences, School of Pharmacy, Virginia Commonwealth University, Richmond, VA, 23298, USA.; 3 Massey Cancer Center, Virginia Commonwealth University, Richmond, VA, 23298, USA.; 4 Institute for Structural Biology, Drug Discovery and Development, Virginia Commonwealth University, Richmond, VA, 23219, USA.

**Keywords:** Circular DNA, theranostics, aptamer, rolling circle amplification, miRNA inhibitors

## Abstract

The past decade has witnessed the blossom of nucleic acid therapeutics and diagnostics (theranostics). Unlike conventional small molecule medicines or protein biologics, nucleic acid theranostics have characteristic features such as the intrinsic ability as “information drugs” to code and execute genetic and theranostic information, ready programmability for nucleic acid engineering, intrinsic stimulatory or regulatory immunomodulation, versatile functionalities, and easy conformational recovery upon thermal or chemical denaturation. Single-stranded circular DNA (circDNA) are a class of single-stranded DNAs (ssDNA) featured with their covalently-closed topology. In addition to the basic advantages of nucleic acids-based materials, such as low cost, biocompatibility, and simplicity of chemical modification, the lack of terminals in circDNA prevents exonuclease degradation, resulting in enhanced biostability relative to the corresponding linear ssDNA. circDNA has been explored for versatile theranostic applications. For instance, circDNA has been extensively studied as templates for bioanalytical signal amplification and the synthesis of nano-/micro-/macro- biomaterials via rolling circle amplification (RCA) and rolling circle transcription (RCT) technologies. circDNA has also been commonly used as the scaffolds for the self-assembly of versatile DNA origami. Finally, circDNA has been implemented as theranostic aptamers, miRNA inhibitors, as well as clustered regularly interspaced short palindromic repeats-CRISPR-associated proteins (CRISPR-Cas) gene editing donors. In this review article, we will discuss the chemistry, characteristic properties, and the theranostic applications of circDNA (excluding double-stranded circular DNA such as plasmids); we will also envision the challenges and opportunities in this research field.

## Introduction

Nucleic acid theranostics have garnered significant interest for various diseases. Among them are the emerging circular nucleic acid theranostics, including circular RNA (circRNA) and single-stranded circular DNA (circDNA) (to be distinguished with double-stranded plasmid DNA). In this review, we will focus on the discussion of single-stranded circDNA theranostics but not circRNAs that have shown unique immunodulatory properties 1 and have been attempted to be developed as vaccines for SARS-CoV-2 2. Single-stranded circDNA is a class of ssDNA featured with the covalently-closed topology. Natural circDNA has been discovered in the genomes in some of the human, animal, and plant viruses. These natural single-stranded circDNAs are found only in viruses that are typically comprised of a small-sized genome (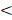
10 kilobases or kb) and capsid proteins without a lipid envelope 3-5. circDNA viruses currently are recognized by the International Committee on Taxonomy of Viruses (ICTV) include seven families: *Anelloviridae*, *Geminiviridae*, *Inoviridae*, *Microviridae*, *Nanoviridae* and *Parvoviridae* that can infect vertebrates, plants and even humans 3-9. circDNA viruses can be monopartite, bipartite or multipartite. The genome organization of these viruses varies between genera with some having ambisense genome organization such as *circoviruses*, whilst others having negative sense genome organization such as* anelloviruses* and *gyroviruses*. Many of the circDNA viruses have been shown to be evolutionarily related in association with the circular replication-associated protein (Rep) gene, which is responsible for initiating viral genome replication via rolling circle replication 10. The single-stranded circDNA viral vectors can be used as vaccines to express recombinant vaccine antigens and monoclonal antibodies. Many current vaccines are derived from the porcine circovirus type 2a (PCV2a) genotype or its capsid protein 11, and are acknowledged as highly successful in decreasing the disease burden found prior to the introduction of the vaccine. The current vaccines are efficient in inducing humoral and cell-mediated immunity against PCV2 12-14. In addition, a long circular single-strand-scaffold viral DNA derived from the bacteriophage M13mp18 can be used as a scaffold to form various shapes of DNA origami, which has revolutionized the field of DNA nanotechnology by enhancing the complexity and size of self-assembled DNA nanostructures in a simple “one-pot” reaction 15.

**Figure 1:**
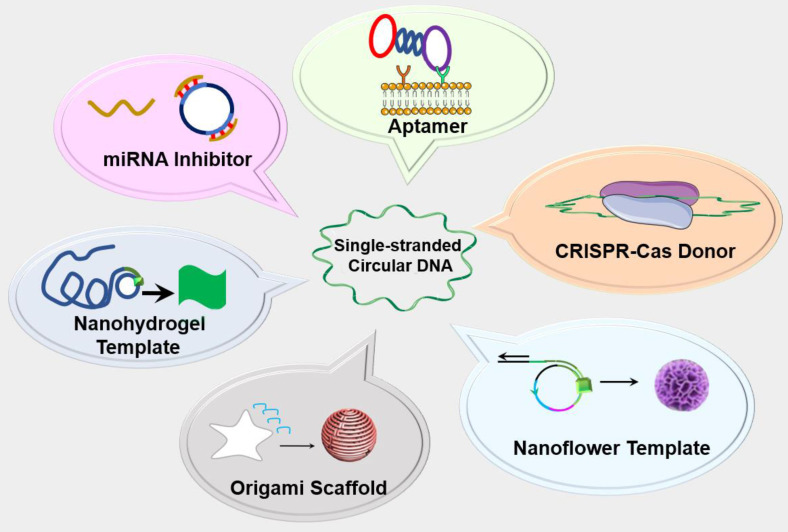
** Schematic depiction of single-stranded circDNA theranostics.** circDNA has been commonly used as theranostic aptamers, templates for DNA nanostructures, scaffolds for versatile DNA origami, miRNA inhibitors, as well as CRISPR-Cas gene editing donors.

In addition to natural circDNA, synthetic circDNA has recently been used for research and drug development 16. Compared to natural circDNA, synthetic circDNA can be synthesized mainly using DNA ligases with customized lengths and sequences for versatile theranostic applications. The lack of terminals in circDNA prevents exonuclease degradation, resulting in enhanced biostability, relative to linear ssDNA. For example, by using the circDNA template, a short DNA or RNA primer can be amplified to form a long single stranded DNA or RNA by rolling circle replication (RCR or RCT) 16. The resulting products are concatemer DNA (for RCR) and RNA (for RCT) containing up to hundreds of tandem repeats that are complementary to the circDNA templates. The simplicity and programmability of these DNA amplification technologies have made them attractive for biomedical research and applications. Moreover, since RCR and RCT products can be tailor-designed via the circDNA templates, RCR and RCT have been employed to generate complex DNA nanostructures such as DNA nanostructures 17, nanotubes 18, nanoribbons 19 and DNA based metamaterials 20. These nanotechnologies have been utilized for bioanalysis, drug delivery, bioelectronic circuits and bioseparation. In addition, theranostics circDNA probes such as aptamers have garnered increasing research momentum for theranostic applications. Lastly, circDNA has been recently applied for genetic manipulation and editing via systems, such as CRISPR-Cas system and microRNA (miRNA) inhibitors. In this review article, we will discuss the design, synthesis methods, as well as versatile theranostics application of circDNA (Figure 1).

## Single-stranded circDNA-based aptamers for theranostic applications

Aptamers are short ssDNA or ssRNA that can selectively bind to specific targets, including proteins, peptides, carbohydrates, small molecules, toxins, and even live cells 21. Aptamers assume unique three-dimensional configurations, which are the basis for the selective binding abilities to cognate targets. Aptamers are typically generated by an evolutionary technology called systematic evolution of ligands by exponential enrichment (SELEX), using an oligonucleotide library with millions of randomized DNA sequences 21. Once DNA aptamer sequences are obtained by SELEX, the aptamers can be chemically synthesized on a large scale for theranostic applications. Linear aptamers are susceptible to exonuclease degradation and may be limited by the intermediate sensitivity of aptamer-based biosensors, Due to the lack of DNA terminus, circDNA aptamers enhance the biostability relative to the corresponding linear DNA aptamers. In addition, to promote the sensitivity of bioanalysis, circDNA aptamer templates and RCR have been employed in aptamer-based biosensors. In this section, we will discuss circDNA aptamers for theranostic applications.

### circDNA aptamers for targeted drug delivery

**Figure 2:**
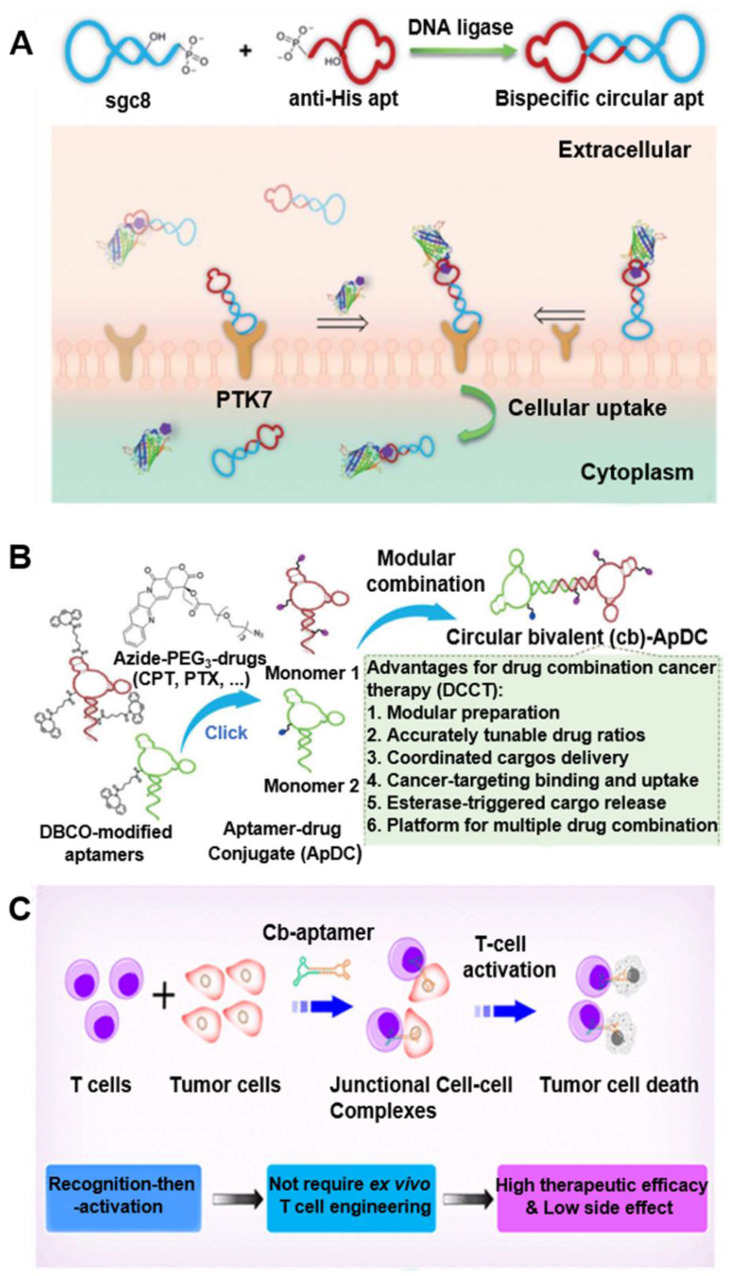
**circDNA aptamers for versatile disease theranostic applications. (A)** Synthesis scheme of bispecific circDNA aptamer for bispecific recognition of His tagged protein cargoes and PTK7 on targeted cells. Reprinted from 23, copyright (2020) The Royal Society of Chemistry Publishing Group. **(B)** Preparation of bivalent circDNA ApDCs with accurate tunability of drug ratios for drug combination cancer therapy (DCCT). Reprinted from 25, copyright (2019) The Wiley-VCH Verlag GmbH & Co. KGaA, Weinheim. **(C)** Schematic illustration of the bispecific circDNA aptamer and (b) its molecular-mediated “recognition-then-activation” for targeted immunotherapy. Reprinted from 26, copyright (2020) The American Chemical Society Publishing Group.

Cyclization of aptamers can improve their thermal stability, nuclease resistance, and binding affinity 22. Further, *in vivo* fluorescence imaging showed the efficient accumulation and retention of circDNA aptamers in tumors relative to linear aptamers, which provides a simple and efficient strategy to improve the pharmacokinetics and hence tumor theranostic efficacy of DNA aptamer. In one example, by cyclization of two Sgc8 aptamer that recognize a cancer biomarker protein tyrosine kinase 7 (PTK7) 22, that the resulting bivalent circDNA aptamer (cb-apt) with two identical cell-specific aptamers showed enhanced nuclease resistance and higher binding ability *in vivo*, relative to monomers. In another example, bispecific circDNA aptamer 23 formed from 1) an anti-His tag aptamer that binds to the biorthogonal six polyhistidine tag (His tag) on functional proteins (Figure 2A) 23, and 2) a cancer biomarker PTK7-specific aptamer Sgc8. The resulting bivalent circDNA aptamer facilitated the targeted delivery of functional proteins into PTK7-positive target cells, improving their overall bioactivity in the cellular milieu by *ca.* 4-fold. Further a β-cyclodextrin (CD)-conjugated bivalent circDNA aptamers facilitated the targeted intracellular delivery of functional proteins that are loaded into β-CD-conjugated circDNA aptamer via host-guest chemistry 24. The resulting circDNA aptamer exhibited high serum stability and enhanced intracellular delivery efficiency compared to a monomeric aptamer. Moreover, to study circDNA aptamer for targeted drug delivery, a bivalent circDNA aptamer-drug conjugates (ApDCs) (Figure 2B) was developed and exhibited high biostability, target specific recognition, excellent cellular uptake, and esterase-triggered drug release 25, making this circDNA ApDCs a promising platform for the development of targeted cancer drug delivery systems. Besides using circDNA aptamers for the recognition of cognate molecules, circDNA aptamers have also been studied to bind to cellular targets. For instance, bispecific circDNA aptamers were constructed from two aptamers that binds to naiv̈e T cells and tumor cells, respectively, resulting in circDNA aptamer that can simultaneously bind to two different types of cells and bring these two types of cells into close proximity (Figure 2C) 26. After T cell accumulation in the tumor mediated by bispecific naiv̈e T cells and tumor cells aptamers, T cells in the tumor site were subsequently activated *in situ* via CD3/CD28 T cell activator to induce tumor killing. By using different cancer-specific aptamers, this “recognition-then-activation” strategy has the potential for targeted treatment of other types of cancer. Finally, in addition to theranostic application in cancer, circDNA aptamer has been studied for targeted drug delivery in the treatment of neurodegenerative disorders. Specifically, a circular bifunctional circDNA aptamer was synthesized from 1) a transferrin receptor (TfR)-targeting aptamer that facilitates TfR-induced transcytosis across the endothelial cells in the blood-brain barrier (BBB), and 2) a Tau protein-specific aptamer that inhibits Tau phosphorylation and tauopathy-related pathological events in the brain. The resulting bifunctional circDNA aptamer enhanced the *in vivo* BBB penetration for tauopathy therapy 27. Collectively, these studies showed the potential of circDNA aptamers to facilitate their theranostic applications.

### Aptamer-integrated circDNA for sensitive bioanalysis

Rolling circle amplification (RCA), such as RCR and RCT, is an isothermal nucleic acid amplification process that synthesizes long single-stranded nucleic acids from continual and unidirectional replication of circDNA templates 16, 28. Thus, RCA can amplify a single molecular binding event by up to three orders of magnitudes, making it ideal for numerous applications that require ultrasensitive detection. These assays usually use immobilized antibodies or aptamers to capture target molecules from sample solutions. Aptamer containing a primer region for annealing to the circDNA of RCA are then introduced to bind to the captured target molecules. Unbound aptamer probes are washed away, prior to RCA. The RCA products are then detected using various signal generation techniques. For instance, on the basis of the structural isolation of two thrombin-binding DNA aptamers, each of which recognizes thrombin at a different location, one of the aptamers was converted to circDNA 29. In the absence of thrombin, the duplex was too weak to form; by contrast, when thrombin was present, both aptamers engaged the same thrombin molecule for binding, which led to the formation of the DNA duplex and triggered the RCA process for signal amplification and hence sensitive detection of thrombin.

**Figure 3:**
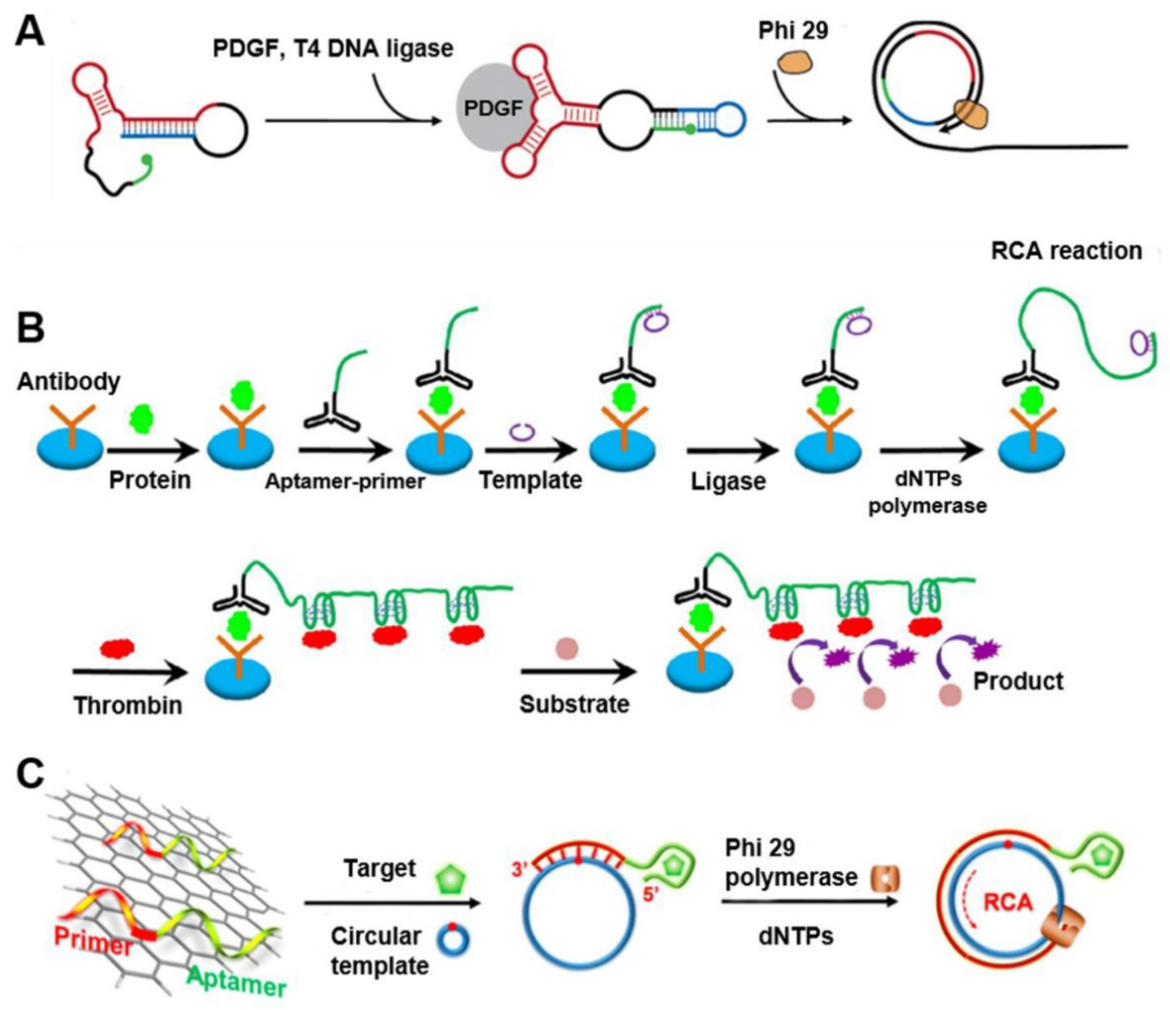
**Aptamer-integrated circDNA for sensitive bioanalysis. (A)** RCA assay using a structure-switching aptamer that circularizes upon target binding. Reprinted from 30, copyright (2007) The American Chemical Society Publishing Group. **(B)** Schematic of the aptamer assay for protein detection using RCA coupled with thrombin catalysis. Protein target is captured by an antibody coated on microplate, and then bound with the conjugate of aptamer-primer in which the aptamer binds to a protein target. The template encoding complementary sequence of the thrombin aptamer hybridizes with the primer, and is circularized by ligase to form circDNA as the templates of RCA. The resulting long DNA generated from RCA binds with multiple thrombin molecules, achieving multiple thrombin labeling in sandwich complex. Thrombin catalyzes the cleavage of small peptide substrates into detectable product. Reprinted from 31, copyright (2016) Springer-Verlag Berlin Heidelberg. **(C)** RCA-mediated assay based on target binding-induced desorption of aptamers from GO. Reprinted from 32, copyright (2014) The American Chemical Society Publishing Group.

Aptamers can also be designed to directly trigger RCA. Yang et al. designed a structure-switching aptamer that can circularize upon target binding (Figure 3A) 30. A 13-nt sequence was added to the 3′-end of an aptamer for platelet-derived growth factor BB (PDGF-BB). This 13-nt sequence was complementary to the aptamer and disrupted the binding secondary structure of the anti-PGDF-BB aptamer by forming a hairpin. The aptamer was further modified by adding a 6-nt sequence to its 5′-end and altering the linker sequence so that target binding resulted in a closed ligation junction. Therefore, enzymatic ligation generated a circDNA that served as an RCA template. This structure-switching aptamer was used for the homogeneous detection of PGDF-BB, achieving a detection limit of 0.4 nM. Another group described a sensitive aptamer-based sandwich assay for protein detection on microplates by using RCA coupled with thrombin catalysis (Figure 3B) 31. This assay takes advantage of RCA to generate long DNA with repetitive thrombin-binding aptamer for multiple thrombin labeling that then promote the enzyme activity of thrombin for signal generation. They applied this strategy for the detection of PDGF-BB as a model protein target. Due to double signal amplifications from RCA and thrombin catalysis, this assay enabled the detection of PDGF-BB as low as 3.1 pM when a fluorogenic peptide substrate was used. In another study, since DNA aptamers can be absorbed on graphene oxide (GO) and be dissociated upon target binding of aptamers, Li and co-workers developed a RCA-mediated homogeneous assay for the amplified detection of proteins and small molecules using GO (Figure 3C) 32. When aptamers comprised of RCA primers are adsorbed on a GO surface, RCA was disabled due to the unavailability of RCA primers. The binding of target molecules to aptamers released the aptamers from GO, thereby exposing the primers for RCA template annealing and RCA initiation. The assay was used for the sensitive detection of thrombin and ATP as model targets.

## circDNA as donors in CRISPR-Cas system

The prokaryotic CRISPR-Cas genome editing systems have transformed our ability to manipulate, detect, image and annotate specific DNA and RNA sequences in living cells of diverse species 33. The ease of use and robustness of this technology have revolutionized genome editing for basic research as well as disease theranostics, among others 33. As a key element of genome editing, donor DNA introduces the desired exogenous sequence while working with other crucial machinery such as CRISPR-Cas or recombinases. While genome editing has been revolutionized by the advent of CRISPR-based nucleases, difficulties in achieving efficient, nuclease-mediated, homology-directed repair (HDR) can limit their application. Commonly used DNA donors such as plasmids suffer from low HDR efficiencies in many cell types as well as integration at unintended genomic sites 34. Consequently, there is considerable interest in developing methods to generate long ssDNA templates as donors for targeted DNA insertions in mammalian cells. Experiments with donor ssDNA yielded between 10 and 100% recombination frequency for point mutations 35. Several recent strategy to generate such ssDNA include asymmetric PCR, “Strandase” enzyme-mediated removal of one strand of a linear dsDNA template, use of pairs of nicking endonucleases followed by gel extraction of resulting ssDNA, and reverse transcription (RT)-based approaches to generate ssDNA 36-38. Most of these approaches require expensive and time-consuming purification to ensure complete removal of truncated ssDNA products. With RT-based approaches in particular, it is challenging to generate accurate ssDNA donors longer than 3-4 kb, especially in large molar quantities, because of the lack of proofreading activity and the limited processivity of reverse transcriptases.

**Figure 4:**
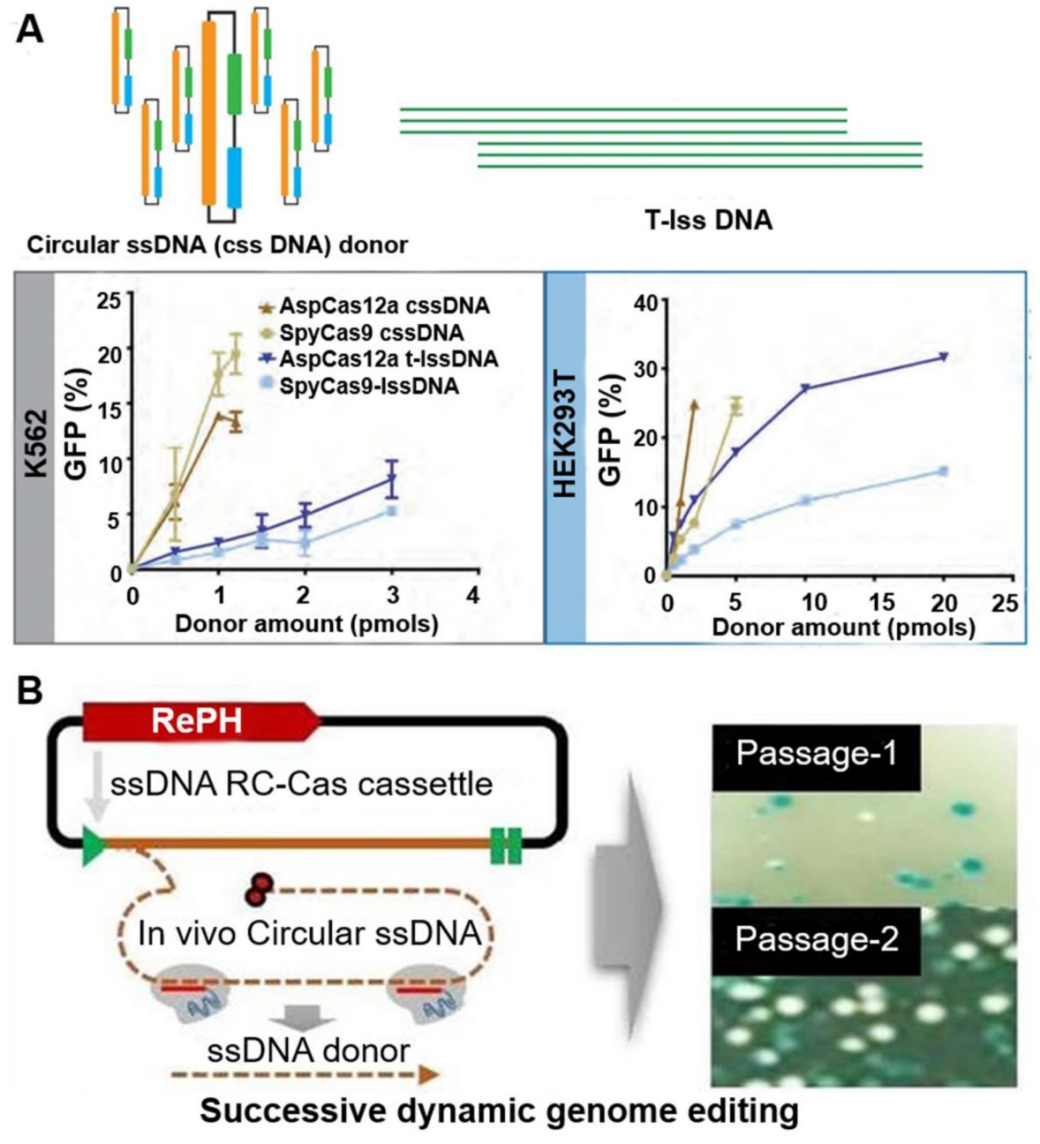
** circDNA as donors in genome editing. (A)** Comparisons of the activity of different DNA donors in HCR using the Traffic Light Reporter Multi-Cas Variant 1 (TLR-MCV1) cassette in human cells. circular ssDNA (cssDNA) and T-lssDNA (lssDNA: linear ssDNA) donor template-mediated HDR efficiency is dose dependent. The graph shows the percentage of GFP-positive cells as a function of increasing cssDNA and T-lssDNA donor DNA in the presence of SpyCas9 and AspCas12a proteins in TLR-MCV1 K562 cells (left) and HEK293T cells (right). Reprinted from 39. **(B)** RCA-Cas-mediated genome editing *in vivo*. The mechanism of the production of linear ssDNA. SpCas9-mediated DNA cleavage of the targeted circDNA, in the absence of a protospacer adjacent motif (PAM) sequence. Right image: photographs of the plates showing the effect of linear ssDNA synthesis for gene editing using pRC22. Passage 1, the first round of culture for introducing the allele substitution (11 bp substitution in the lacZ gene); Passage 10, the tenth round of culture for introducing the allele substitution (11bp substitution in the LacZ gene). Reprinted from 40, copyright (2020) The MDPI Publishing Group.

Alternatively, Mir *et al.* used single-stranded circDNA produced from phagemids as templates for HDR-mediated integration of DNA cassette 39. These circDNA donors serve as efficient HDR templates when used in CRISPR-Cas9 and CRISPR-Cas12a, with integration frequencies superior to linear ssDNA donors (Figure 4A). They then extended the analysis of DNA donor to evaluate efficiencies of fluorescent tag knock-ins at endogenous sites in HEK293T and K562 cells. Their results show that circDNA donors resulted in efficient and robust insertion of reporter tags. circDNA donors also outcompeted linear ssDNA donors in template-driven repair at the target site. These data demonstrate that circDNA donors provide an efficient, cost-effective method to achieve knock-ins via CRISPR-Cas gene editing in mammalian cell lines. In another example, Qi *et al.* utilized RCR- and Cas9- mediated *in vivo* ssDNA synthesis (Figure 4B) 40. A single-gene RCR from Gram-negative bacteria was engineered to produce single-stranded circDNA from a Gram-positive parent plasmid at a designed sequence in *Escherichia coli*. Furthermore, the desired linear ssDNA fragment can be cut out using CRISPR-Cas9 and combined with lambda Red recombinase as donor for precise genome engineering. These studies demonstrated the application of circDNA in genome editing.

## circDNA as templates for the synthesis of multiscale biomaterials for versatile biomedical applications

**Figure 5:**
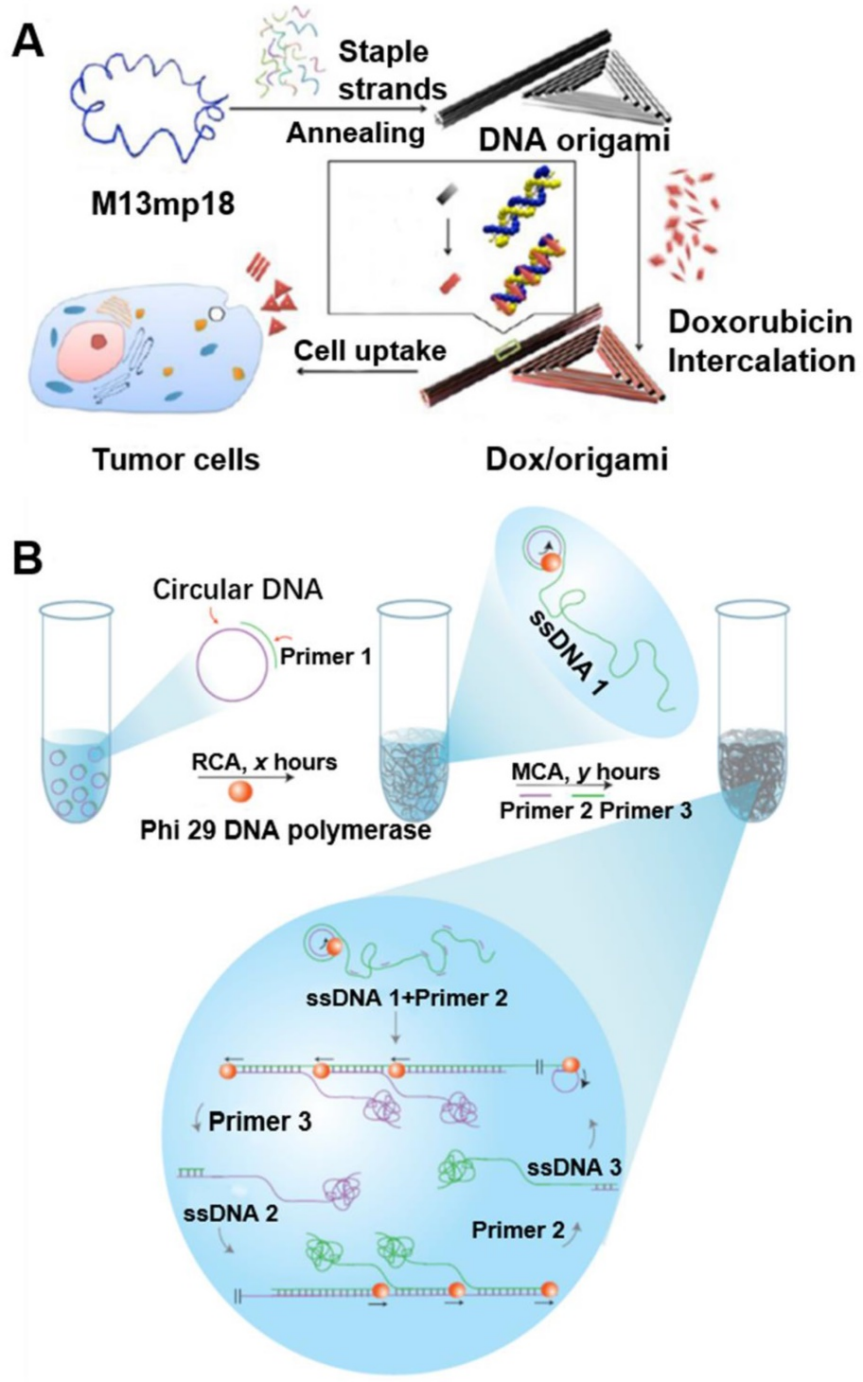
**circDNA as templates to synthesize multiscale (nano-, micro-, and macro-) DNA biomaterials. (A)** Doxorubicin-loaded DNA origami for drug delivery. The long single-strand M13mp18 genomic circDNA scaffold (blue) was folded into the triangle and tube structures through the hybridization of rationally designed DNA staple strands. Doxorubicin was loaded into DNA origami via dsDNA intercalation. Reprinted from 43, copyright (2012) The American Chemical Society Publishing Group. **(B)** Schematic diagram of the stepwise approach for DNA hydrogel synthesis. The multi-step RCA processes were carried out as follows: (i) In the R process, a circDNA template was first produced, then a complementary primer for RCA (Primer 1) was added to produce elongated ssDNA products (termed ssDNA 1: tandem repeats of the sequences complementary to the original circular ssDNA template), and (ii) In the M process, after RCA, two additional primers (Primer 2 and Primer 3) were added for subsequent chain amplification. Reprinted from 20, copyright (2012) The Nature Publishing Group.

DNA nanotechnology has been widely investigated for various theranostic applications 41, such as intracellular delivery of nucleic acid theranostics 42. However, naked DNA cannot penetrate the cell membrane and are prone to be degraded by nucleases in serum or cytoplasm 42. Nanobiotechnology has provided unprecedented opportunities for the delivery of versatile theranostic agents, including nucleic acids. DNA nanostructures have recently been employed as drug delivery carriers 41. In this section, we will discuss the application of circDNA as templates to synthesize DNA biomaterials, such as DNA origami, DNA nanoflowers, and DNA hydrogel (Figure 5).

### DNA origami

DNA origami has revolutionized the field of structural DNA nanotechnology by enhancing the complexity and size of self-assembled DNA nanostructures in a simple “one-pot” reaction 44. In DNA origami, a long circDNA scaffold is folded into various shapes by hundreds of synthetic staple DNA that bind to different sites along the circDNA scaffold, yielding DNA origami nanostructures with well-defined sizes and morphologies 15. For example, various origami can be programmably self-assembled from a circDNA scaffold of bacteriophage genome M13mp18 comprised of 7249 nucleotides, as well as hundreds of short staple DNA 45. Although many different DNA origami nanostructures were synthesized, the increasing need for more DNA origami structures was limited by the fixed sequence and size of the scaffold. To broaden the biomedical application of DNA origami, PCR and genome tailoring technique have been studied for the scaffold design 46. Moreover, DNA origami has been used for the preparation of different 2D and 3D nanostructures and for the nanopatterning of nanoparticles, proteins and other functional molecular components into well-defined arrangements 45. Chemically-modified DNA staples can be inserted at a predefined position in DNA origami, whereby imparting additional functionalities in DNA origami. DNA origami offers greater flexibility in the functionality and likely great biostability 43, 47. For instance, using immunostimulatory CpG DNA as a model drug, DNA nanoribbons can be loaded with CpG through DNA hybridization for intracellular delivery of CpG. As a result, DNA nanoribbons modified with multiple CpG motifs exhibited a higher immunostimulatory effect, as indicated by the efficient production of the inflammatory cytokines tumor necrotic factor alpha 41, 47-49. Similarly, spatially addressable DNA origami nanostructures have been studied as drug carrier system (Figure 5A) 43. With DNA origami as drug delivery vehicles, the cellular internalization of doxorubicin was increased, which contributed to the significant enhancement of cell-killing activity to doxorubicin-resistant MCF-7 cells. These results suggest the potential of DNA origami as an efficient drug delivery vehicle.

### DNA nanoflowers (NFs)

**Figure 6:**
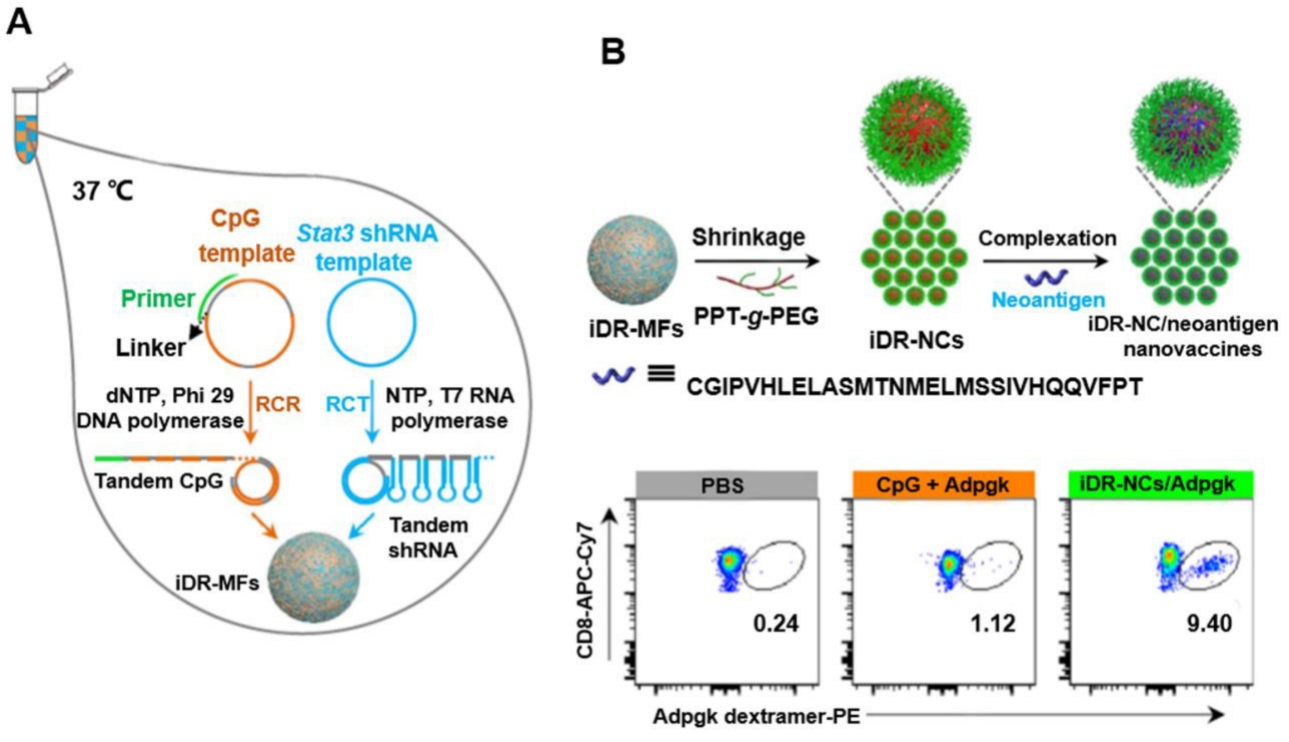
** circDNA-mediated synthesis of DNA NFs for synergistic tumor immunotherapy. (A)** Concurrent RCR and RCT in the same solution generated tandem CpG and Stat3 shRNA, which were self-assembled into intertwining DNA-RNA MFs. **(B)** The above MFs were shrunk by PPT-g-PEG to form iDR-NCs, which was further loaded with tumor-specific neoantigen via hydrophobic interactions between peptide antigens and hydrophobic PPT moieties. **(C)** iDR-NC/neoantigen elicited potent and durable neoantigen-specific T cell responses. Representative flow cytometry of neoantigen ASMTNMELM-specific CD8^+^ T cells among live (DAPI^-^) CD8^+^ cells in PBMCs, as stained using a PE-conjugated ASMTNMELM-H-2D^b^ dextramer. Reprinted from 56, copyright (2015) The Nature Publishing Group.

DNA and pyrophosphate released from polymerase reactions such as RCA can form compact organic-inorganic composite NFs 50. In contrast to the key role of DNA base pairing in DNA nanostructures formation, NF assembly is independent of DNA hybridization. In addition, unlike other DNA nanostructures, NFs have unique interior and exterior morphologies (Figure 6) 51. NFs can be assembled using DNA building blocks by RCA (i.e., RCR and RCT) using circDNA templates, optional DNA primers 52. Due to the dense DNA packaging, NFs resist to nuclease degradation, dissociation, or thermal or chemical denaturation. Recently, NF blooming has been used to design a simple and sensitive electrochemical biosensing system. In this system, the NF blooming in the nanochannels has been tailored to occur upon binding of target miRNA, followed by binding of a circDNA template and a specific primer to the captured miRNA, triggering RCA and NF blooming in the nanochannels. NF formation increased steric hindrance in the channels, which reduced the anodic current of potassium ferricyanide (K_3_[Fe(CN)_6_]), thus enhancing electrochemical detection signals 51. In addition to the excellent biostability, NFs exhibit excellent photostability, as shown by aptamer-conjugated multicolor fluorescence resonance energy transfer (FRET)-NFs 53. Moreover, immuno-nanoflowers have been developed for nuclease resistance and efficient CpG delivery 54. CpG NFs were found to be potent immunostimulators that stimulated a variety of cells to secrete proinflammatory cytokines. Moreover, pH-responsive multifunctional DNA NFs containing cancer-targeting aptamer, fluorophores, and Dox molecules were used to prevent drug efflux and improve drug retention in MDR cells, thus bypassing MDR (Figure 6) 52, 55. In addition, using NF nanosystem, synergistic nanovaccines were constructed for cancer immunotherapy. The NFs were formulated to comprise of intertwining DNA-RNA nanocapsules (iDR-NCs) conjugated with DNA CpG, *Stat3*-targeting short hairpin RNA (shRNA), as well as tumor-specific peptide neoantigens. shRNA in iDR-NCs synergistically activated antigen-presenting cells (APCs), primed neoantigen-specific CD8+ T cells, induced T cell memory, and markedly suppressed the progression of neoantigen-specific colorectal tumors 56.

### DNA hydrogel

The large DNA sizes of the RCA products make it a well suited building block to construct nano-, micro- and even macroscale biomaterials. Luo and co-workers have reported a DNA metastable hydrogel made of 3D entangled RCA DNA products 20. Remarkably, these new metamaterials possess unique mechanical properties as they behave either liquidlike or solid-like depending on the physical environment. Specifically, the gel remains in solid-like form in water while flowing freely in a ''liquid'' form when it is taken out of water. The liquid form of the hydrogel can adopt different shapes of the container, and interestingly, it recovers rapidly to its original shape when returned to water, regardless of how many different shapes it had adopted in the liquid-like state. Specifically, the DNA hydrogel is formed due to DNA entanglement resulting from RCA couple with multi-primed chain amplification (MCA). The hierarchical structure of the DNA hydrogel, which is attributed to the above-mentioned mechanical properties, can be tuned by changing the reaction time of RCA and MCA. These unique metaproperties of the DNA hydrogel may find applications in drug release, cell therapy, electric switches and flexible circuits (Figure 5B) 20.

## circDNA as miRNA inhibitors

**Figure 7:**
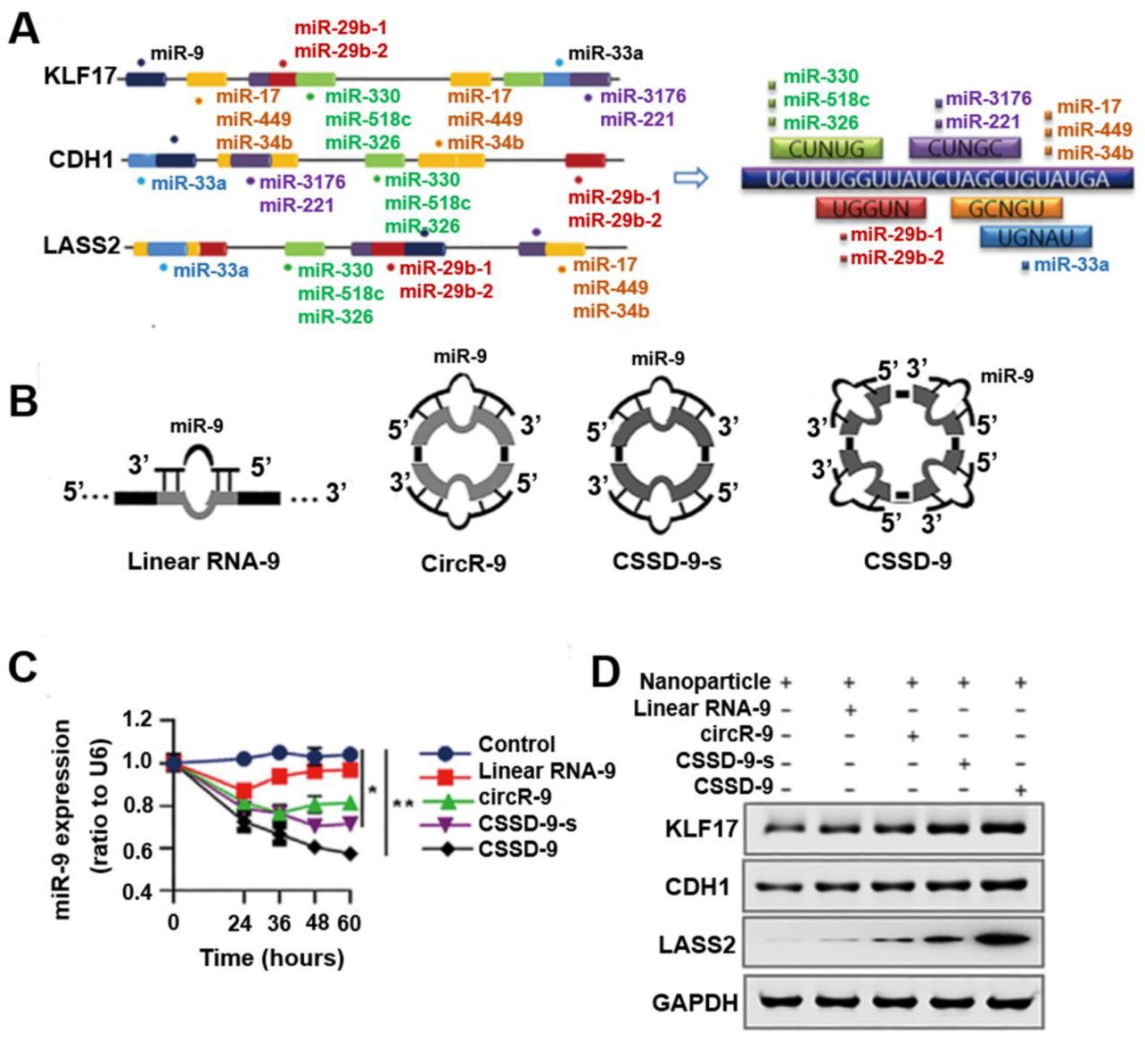
** Nanoparticle-loaded single-stranded circDNA served as a miR-9 sponge that increased tumor suppressor gene expression and inhibited tumor proliferation and metastasis. (A)** Motifs from miRNAs targeting KLF17, CDH1, and LASS2 found by a database search. **(B)** Several miR-9 sponges (linear RNA-9, circR-9, CSSD-9-s, and CSSD-9) were designed. **(C)** qRT-PCR detection of miR-9 expression in HeLa cells after transfection of nanoparticles with different miR-9 sponges for different time points. miR-9 expression was normalized by reference gene U6 small nuclear RNA expression. **(D)** Western blot analysis of KLF17, CDH1, and LASS2 proteins in HeLa cells transfected with nanoparticles with different miR-9 sponges. Reprinted from 66, copyright (2018) The Science Publishing Group.

miRNAs are an evolutionarily conserved class of small regulatory noncoding RNAs, binding to complementary target mRNAs and resulting in mRNA translational inhibition or degradation, and they play an important role in regulating many aspects of physiologic and pathologic processes in mammalian cells 57. Epigenetic dysregulation and somatic mutations leading to the silencing of multiple tumor suppressor genes are prevalent in tumors. The dysregulation of miRNAs has been implicated in tumor malignancy, and each miRNA can have multiple target genes 58, 59. Therefore, regulating the expression of even a single miRNA to simultaneously release the co-silencing of multiple tumor suppressor genes may be an effective tumor therapy. While most miRNA inhibitors are based on antisense molecules to bind and sequester miRNAs from their natural targets, it is challenging to achieve effective and stable miRNA inhibition 57. Recently, circular RNAs (circRNA) enriched with miRNA binding sites were observed to act as natural miRNA sponges 60, 61, by which they can regulate gene expression 62, 63. CircRNA MTO1 functions as a sponge of miR-9 to suppress hepatocellular carcinoma progression 64, and silencing of the endogenous circRNA, circHIPK3, and inhibited human tumor cell growth by sponging multiple miRNAs 65. Because circRNAs resists exonuclease degradation relative to the linear RNA counterparts, circRNAs may absorb miRNAs more effectively than linear miRNA inhibitors. These observations suggested the potential of engineered circular nucleic acids rich in miRNA binding sites to inhibit miRNA function and prevent tumor growth. Based on this, by mimicking circRNA characteristics, Yang et al. developed an artificial single-stranded circDNA molecule containing continuous, multiple miR-9 complementary sites that are more stable and resistant to degradation (Figure 7) 66. Their results showed that single-stranded circDNA effectively released multiple, co-silenced tumor suppressor genes (*KLF17*, *CDH1*, and *LASS2*), up-regulating their expression by absorbing oncogenic miR-9 and consequently suppressing tumor progression and lung metastasis. Taken together, single-stranded circDNA has four miRNA binding sites, with each binding site including a bulged site. The imperfect miRNA sponges were attributed to the impediment of quick turnover of the sponge by endonucleolytic cleavage, which prolonged the sequestering effects of the miRNA. The results showed that single-stranded circDNA was more efficient and stable than other linear or cyclic miRNA sponges. These findings revealed that single-stranded circDNA as a miRNA inhibitor can effectively decreased tumor-promoting miRNA activities by transfecting nanoparticles *in vitro* and *in vivo*.

## Conclusion

Nucleic acid theranostics have unique characteristics compared to conventional small molecular theranostics and other biologics such as proteins and peptides. The past few decades have witnessed the development of multiple novel nucleic acid theranostic strategies, established a plethora of nucleic acid chemistry, and developed formulations and delivery systems for the efficient delivery of various nucleic acid theranostic agents. circDNAs are attractive to facilitate the development of DNA theranostics for versatile applications such as targeted drug delivery via circDNA aptamers, biomarker analysis using circDNA aptamers, gene editing using circDNA donor, and biomaterial development for bioanalysis and delivery of nucleic acid therapeutics and vaccines. Of note, the advancement of these circDNA theranostics can be further fueled by a plethora of well-established nucleic acid chemistries and preexisting nucleic acid delivery systems that enhance their biostability, improve their *in vivo* pharmacokinetics and pharmacodynamics, and promote their theranostic efficacies. An increasing number of nucleic acid theranostics have been approved by the FDA or equivalent agents around the world. Relative to conventional theranostics, the development of circDNA theranostics have unique challenges and opportunities. So far, the relatively high cost for large-scale DNA manufacturing remains challenging, which may impede their broad applications especially in low-income settings. Further, the efficiency of targeted delivery of nucleic acids to many organs, except for liver and spleens, remains suboptimal. Meanwhile, the past experiences of currently FDA approved nucleic acid theranostics would facilitate the development of circDNA theranostics. Overall, circDNA holds great potential for versatile applications in the theranostics of various diseases.
